# Myxoid liposarcoma of bladder: a rare case

**DOI:** 10.1016/j.ijscr.2020.12.088

**Published:** 2021-01-09

**Authors:** Sampanna Chudal, Sujeet Poudyal, Suman Chapagain, Bhoj Raj Luitel, Pawan Raj Chalise, Uttam Kumar Sharma

**Affiliations:** Department of Urology and Kidney Transplant Surgery, Tribhuvan University Teaching Hospital, Institute of Medicine, Maharajgunj, Kathmandu, 44600, Nepal

**Keywords:** Myxoid liposarcoma, Bladder sarcomas, Genitourinary sarcomas

## Abstract

**Introduction:**

Myxoid liposarcoma of the bladder is a rare mesenchymal tumor which is difficult to diagnose and carries a poor prognosis. The primary treatment is complete surgical resection which is usually followed by adjuvant chemotherapy.

**Presentation of case:**

We report a case of 26 years female who presented with a large abdominal mass and was initially misdiagnosed to be arising from ovaries and subsequently discovered to be a mesenchymal tumor arising from the bladder.

**Discussion:**

Myxoid liposarcoma is an exceedingly rare but aggressive mesenchymal tumor of the bladder often with misleading imaging appearances.

**Conclusion:**

Surgical resection, when feasible, plays the leading role in its management, and should be attempted in all potential patients.

## Highlights

• Myxoid liposarcoma is a rare mesenchymal tumor of the bladder with only a few cases reported in the literature.
• It is difficult to diagnose and carries a poor prognosis.
• The primary treatment is complete surgical resection which is usually followed by adjuvant chemotherapy.
• We report a case of 26 year female with mesenchymal tumor of the bladder presenting as a large abdominal mass.

## 1 Introduction

Myxoid liposarcoma is a rare mesenchymal tumor of the bladder with only few cases reported in the literature [1,2]. The diagnosis is usually not straightforward and is complicated by factors such as atypical presentation, misleading imaging findings and rarity of the tumor itself. Prognosis is poor owing to the aggressive nature of the tumor. Surgical resection remains the mainstay of treatment and should be attempted whenever feasible.

This case report has been reported in line with the SCARE Criteria [3].

## 2 Case report

A 26 year female presented with lower abdominal pain and distension for 1 month. She did not have any lower urinary tract symptoms or hematuria. She did not have any significant past medical or surgical history. On abdominal examination, there was a firm palpable mass in the lower abdomen with restricted mobility. General physical examination and systemic examination was essentially normal.

**Fig. 1:**
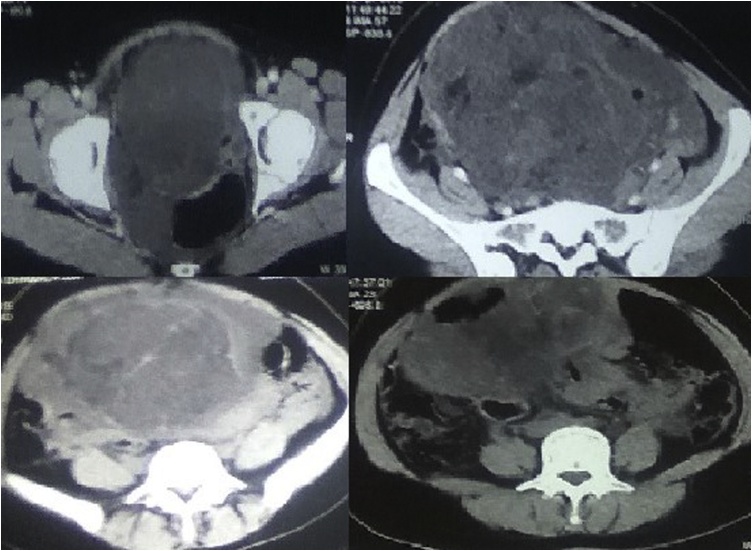
CECT abdomen/pelvis showing a large mass in the pelvis extending to lower abdomen.

Upon ultrasonic examination, a large heterogeneous mixed echoic abdominopelvic mass approximately 17.7 × 13.7 cm was visualized arising from pelvis and extending to upper abdomen. Uterus was not separately visualized. CECT abdomen/pelvis revealed approximately 21.7 × 15.4 × 15 cm sized ill-defined heterogeneous mass with fat component and hemorrhage in abdominopelvic cavity (Fig. 1) reaching superiorly up to the level of umbilicus. The left ureter was compressed by the mass causing mild hydronephrosis. Right kidney was atrophic. There was loss of fat plane with uterus and bilateral ovaries were not separately visualized.

**Fig. 2:**
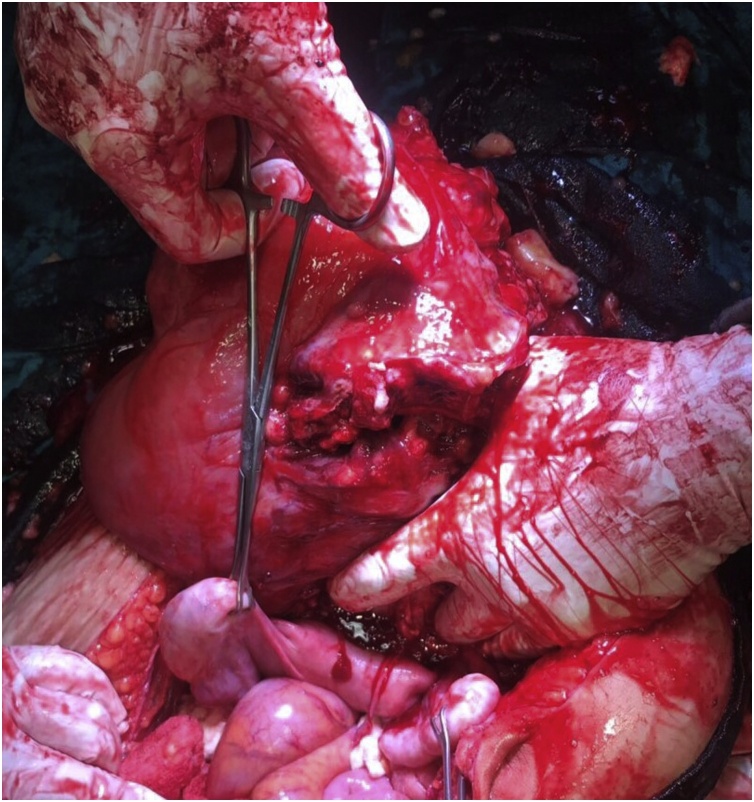
Intraoperative photograph showing huge mass arising from pelvis.

**Fig. 3:**
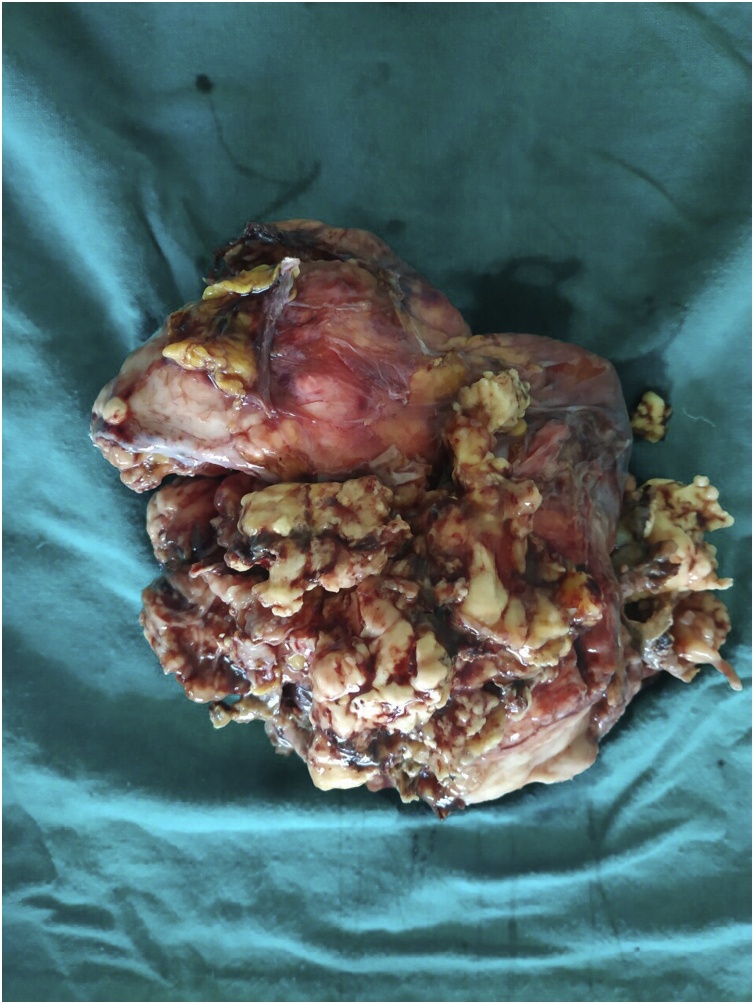
Specimen photograph showing myxoid nature of the mass.

A provisional diagnosis of immature teratoma was made based on imaging findings and patient was taken up for hysterectomy. Cystoscopy was done at the time of surgery which showed bulge in the posterior bladder wall with normal bladder mucosa. On exploration, a huge mucinous mass was seen involving pelvic wall and adherent to posterior urinary bladder wall (Fig. 2, Fig. 3). Left distal ureter was encased within the mass. Anterior pelvic exenteration was done and left ureterocutaneostomy was made for urinary drainage.

**Fig. 4:**
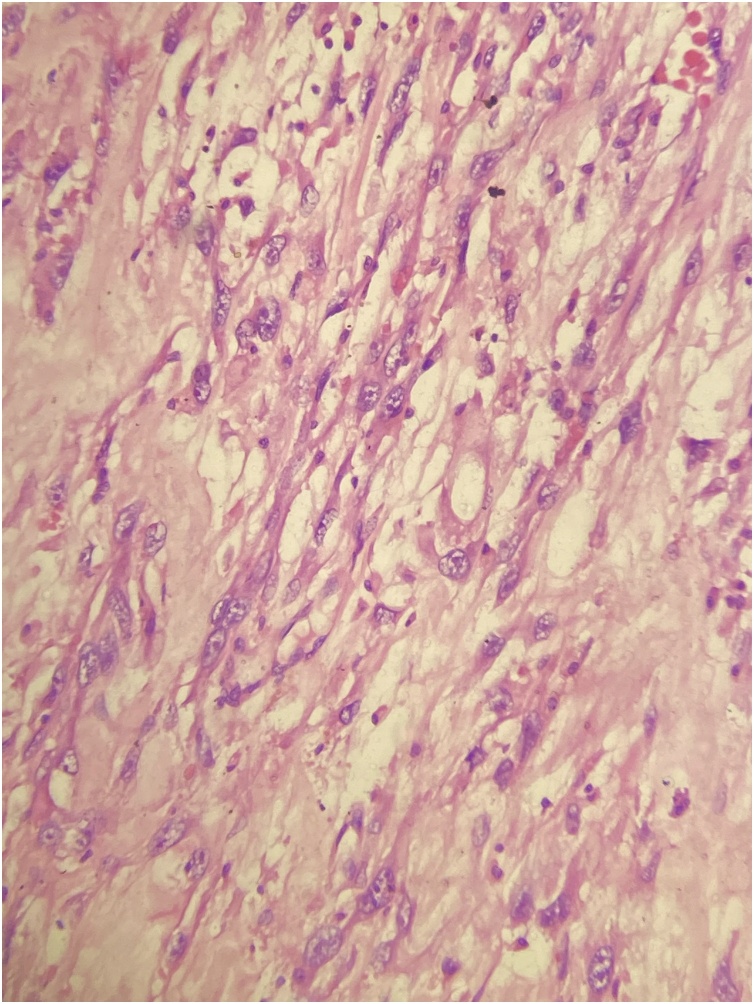
Histopathologic image showing tumor cells interspersed in a myxoid background.

Postoperative period was uneventful and DJ stent was removed after 2 weeks. Histopathological examination revealed the mass to be myxoid liposarcoma arising from bladder wall involving perivesical fat, uterus and cervix (Fig. 4). The patient was then referred for chemotherapy to medical oncologist.

## 3 Discussion

Sarcomas are the most common mesenchymal tumors of the bladder. However, they account for only less than 1% of bladder tumors overall as most bladder tumors are of urothelial origin. These tumors occur twice more frequently in females and are mostly seen in the sixth decade of life. Among bladder sarcomas, the most common histologic subtype is leiomyosarcoma followed by rhabdomyosarcoma, angiosarcoma, osteosarcoma and carcinosarcoma. Myxoid liposarcoma is very rare tumor of bladder with only a few cases reported in the literature. First case was reported by Rosi et al. in 1983 in a 36 year old male presenting with hematuria [1].

Majority of bladder sarcomas are high grade tumors presenting mostly with gross painless hematuria (79%) and local irritative symptoms (16%). Transurethral resection of the tumor is essential in making a diagnosis. Tumors localized to the bladder are managed with radical cystectomy. Achieving negative surgical margins during resection is particularly important as local recurrence rate is 2.4 times higher in patients with positive surgical margins [4]. Chemotherapy though not highly effective is routinely employed given the aggressive nature of tumors and mostly includes doxorubicin, ifosfamide and cisplatin [5].

In our case, preoperative diagnosis was made difficult by numerous factors. The patient was a young female with no urinary tract symptoms and imaging findings pointed toward a ovarian mass. Diagnosis was suspected during surgery due to typical gross appearance of the tumor and confirmed with histopathology.

## 4 Conclusion

Myxoid liposarcoma is an exceedingly rare but aggressive mesenchymal tumor of the bladder often with misleading imaging appearances. Surgical resection, when feasible, plays the leading role in its management, and should be attempted in all potential patients.

## Declaration of Competing Interest

No conflicts of interest to disclose.

## Funding

None.

## Ethical approval

The study was exempt from ethical approval as it was a case report.

## Consent

Written informed consent was obtained from the patient for publication of this case report and accompanying images without disclosure of identity. A copy of the written consent is available for review by the Editor-in-Chief of this journal on request.

## Author’s contribution

Sampanna Chudal: Writing the paper, Data collection.

Sujeet Poudyal: Data collection.

Suman Chapagain: Data collection.

Bhoj Raj Luitel: Data collection.

Pawan Raj Chalise: Study concept or design.

Uttam Kumar Sharma: Study concept or design.

## Registration of research studies

Not Applicable.

## Guarantor

Sampanna Chudal.

## Provenance and peer review

Not commissioned, externally peer reviewed.

## Note

No patient or author details are included in the figures.
